# Formulation and Development of a Herbal Antifungal Gel Containing Origanum vulgare and Syzygium aromaticum Essential Oils Against Oral Candida albicans

**DOI:** 10.7759/cureus.54348

**Published:** 2024-02-17

**Authors:** Pavithra Jayasankar, Manjula M Awatiger, Rubina Mulla, Bhaskar Kurangi, Shahana Shahapuri, Deepa R Mane

**Affiliations:** 1 Oral and Maxillofacial Pathology and Oral Microbiology, KLE Vishwanath Katti Institute of Dental Sciences, KLE Academy of Higher Education and Research, Belagavi, IND; 2 Microbiology, KLE Academy of Higher Education and Research, Jawaharlal Nehru Medical College (JNMC), Belagavi, IND; 3 Pharmaceutics, KLE College of Pharmacy, KLE Academy of Higher Education and Research, Jawaharlal Nehru Medical College (JNMC), Belagavi, IND; 4 Cell Culture, Dr. Prabhakar Kore Basic Science Research Center, KLE Academy of Higher Education and Research, Jawaharlal Nehru Medical College (JNMC), Belagavi, IND

**Keywords:** gel, syzygium aromaticum, origanum vulgare, combination therapy, synergism, antifungal effect, oral candidiasis, essential oil, minimum inhibitory concentration, antifungal herb

## Abstract

Background

Oral candidiasis is the most prevalent oral fungal infection, and existing antifungal agents have side effects such as drug intolerance, resistance, and toxicity. Herbal essential oils are emerging as an alternative therapeutic approach for treating fungal infections*. Origanum vulgare *(*O. vulgare*), commonly known as oregano, and *Syzygium aromaticum* (*S. aromaticum*), commonly known as clove, are known to have antifungal properties and are effective against fluconazole-resistant strains. A combination of essential oils has a synergistic effect and aids in achieving effective antifungal activity at sufficiently low concentrations, which could lead to reduced side effects and resistance.

Aim of the study

This study aimed to formulate and develop an herbal antifungal gel containing *O. vulgare *and *S. aromaticum* and evaluate its synergistic antifungal efficacy against oral *Candida albicans* (*C. albicans).*

Methodology

Minimum inhibitory concentration (MIC) and minimum fungicidal concentration (MFC) determinations of *O. vulgare *and *S. aromaticum *essential oils were performed individually and in combination to assess the antifungal activity against *C. albicans. *Based on the obtained MIC and MFC of essential oils in combination*, *an herbal antifungal gel was formulated. Further, to determine the biocompatible nature of the gel, a 3-(4,5-dimethylthiazol-2-yl)-2,5-diphenyltetrazolium bromide (MTT) assay was performed.

Results

We found that a combination of *O. vulgare* and *S. aromaticum* essential oils showed antifungal activity at a lesser concentration, with a MIC of 0.19 *μ*l/ml and MFC of 0.39 *μ*l/ml when compared to their individual concentrations. Based on our results, an antifungal herbal gel comprising a concentration of 0.6 *μ*l/ml of both essential oils was developed to achieve synergistic antifungal activity against oral *C. albicans. *The MTT assay of the herbal gel did not show any cytotoxicity.

Conclusion

The novel herbal antifungal gel containing *O. vulgare *and *S. aromaticum* is biocompatible in nature and provides an alternative therapeutic approach for treating oral candidiasis.

## Introduction

*Candida* species are normal commensals of the human oral cavity and also opportunistic microbes involved in the most prevalent oral fungal infections, such as candidiasis [1]. Around 150 species of *Candida* have been identified, with *Candida albicans* (*C. albicans*) being the chief causative organism for oral candidiasis and accounting for up to 95% of total reported cases [2].

When the host's defenses are weak, *C. albicans* can turn pathogenic and cause a variety of diseases, including the mucosa as well as the whole human body system [3]. Oral candidiasis, also known as “thrush,” is marked by the overgrowth of fungal organisms and infiltration of the overlying superficial tissues, including the tongue, palate, and other oral mucosal areas. Around 30 to 60% of adults and 45 to 65% of infants harbor *Candida *species in their oral cavity [4] and are more likely to manifest in elderly individuals, denture wearers, people suffering from diverse systemic diseases such as diabetes mellitus, oral cancer, immunodeficiency, and patients on long-term antibiotics, steroid therapy, and chemotherapy [5].

Various strategies are known to fight oral candidal infections, including systemic and topical antifungals with fungistatic or fungicidal action [6]. Consumption of the effective dose of these antifungal agents cannot be tolerated due to side effects caused by the use of higher drug concentrations and prolonged therapies. This leads to increased drug resistance, toxicity, and tolerance, making oral fungal infections a serious clinical problem. Resistance to widely used antifungal drugs has also been reported and poses a new challenge; therefore, an alternative therapeutic approach for oral fungal infection management is necessary [7].

Essential oils derived from plants are gaining increased focus for antifungal drug development as they can provide alternative therapeutic approaches because of their accessibility, minimal cytotoxicity, environmental friendliness, and improved biodegradability when compared to standard antifungal drugs [8-9].

Various studies have shown that herbal essential oils exhibit antifungal activity through several mechanisms, such as cell death, disruption of the cell membrane, and subsequent cellular contents leakage [9-10]. *Origanum vulgare *(*O. vulgare*), commonly known as oregano which belongs to the *Lamiaceae *family, has shown high antifungal activity against *C. albicans*, as they contain carvacrol and thymol as their main constituents [11-12]. These phenolic compounds directly inhibit germination and hypha formation in *Candida *[13].

*Syzygium aromaticum* (*S. aromaticum*), commonly known as clove, belongs to the *Myrtaceae *family. *S. aromaticum* is a dried flower bud and has been used for many medicinal purposes due to the presence of main constituents such as eugenol [14-15]. The antifungal activity against pathogenic fungi has been proven by various studies and has shown effective antifungal properties, including fluconazole-resistant strains [13].

Combinations of essential oils produce a synergistic effect that enables the achievement of potent antifungal action at sufficiently low doses of the active ingredients, which could lead to decreased side effects and also help to overcome resistance [12,16]. Thus, the combination of essential oils can be used topically to treat oral fungal infections caused by *C. albicans*.

To date, no studies are available in the literature that have explored the possible synergistic antifungal activity against *C. albicans*. Hence, our present study aims to formulate and develop an herbal antifungal gel containing *O. vulgare *and *S. aromaticum* essential oils and to evaluate its antifungal efficacy against oral isolates of *C. albicans*.

## Materials and methods

The present in-vitro observational study was conducted after obtaining institutional ethical clearance with the Institutional Review Board number (IRB no. 1544).

Procurement of essential oils

Commercially available *O. vulgare* (Chemical Abstracts Service (CAS) number: 8007-11-2) and *S. aromaticum* (CAS number: 8000-34-8) essential oils were procured by steam distillation method from VedaOils manufacturer, India. The essential oils were carefully handled with the laboratory safety precautions mentioned in the data extraction sheet provided by the manufacturer.

Minimum inhibitory concentration (MIC) and minimum fungicidal concentration (MFC) are assessed individually and in combination to assess the antifungal activity against *C. albicans*

**Figure 1:**
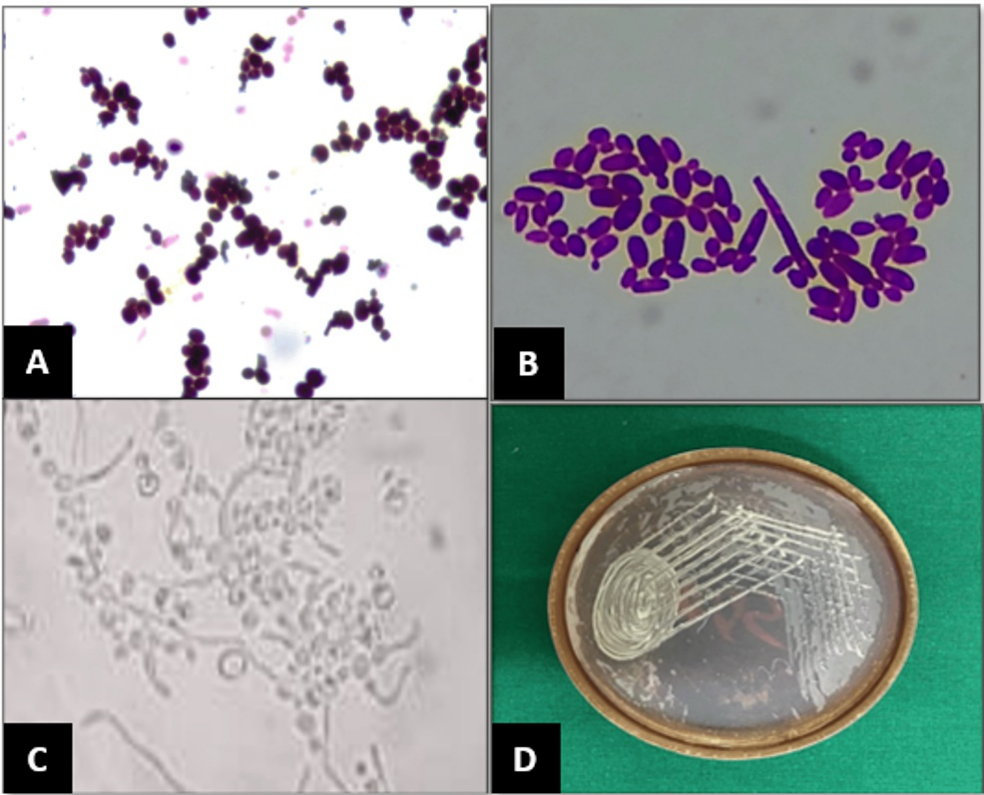
Photomicrograph of C. albicans identification (A) gram’s stain, (B) PAS stain, (C) germ tube test, and (D) standard *C. albicans* strain (ATCC 90028) on SDA plate PAS: periodic acid-Schiff, SDA: Sabouraud's dextrose agar, *C. albicans*: *Candida albicans*, ATCC: American Type Culture Collection

The MIC and MFC of *O. vulgare *and *S. aromaticum* essential oils against oral *C. albicans* were done using standard *C. albicans* strain American Type Culture Collection (ATCC) (90028). Gram staining, periodic acid-Schiff staining, KOH staining, and germ tube testing were done for identification and confirmation. Further, the isolation and culture of *C. albicans* in Sabouraud’s dextrose agar (SDA) medium were performed (Figure 1).

Evaluation of the MIC and broth MFC of *O. vulgare *and *S. aromaticum* essential oils, individually and in combination at various dilutions, was done using SDA and 1% dimethyl sulfoxide (DMSO) with a standard antifungal drug (fluconazole). *C. albicans* was grown and subcultured on an SDA medium, and further MIC and MFC were determined according to the Clinical Laboratory Standards Institute guidelines [17]. A fresh culture after 48 hours was used for the experiment and was grown in SDA broth. The optical density of the suspension was adjusted to 1.5 × 10^8^ colony-forming units/ml (McFarland 0.5 standard).

The essential oils were serially diluted in test tubes with a volume of 5000 μl of 1:10 dilution, which consisted of 4500 μl of 1% DMSO and 500 μl of oil (*O. vulgare* and *S. aromaticum* oils). Along with it, a positive control with 500 μl of emulsion and 500 μl of 1% DMSO and a negative control with 500 μl of essential oil and 500 μl of 1% DMSO were also taken (Table 1).

**Table 1:** Serial dilutions to determine the MIC and MFC MIC: minimum inhibitory concentration, MFC: minimum fungicidal concentration

	Tube 1	Tube 2	Tube 3	Tube 4	Tube 5	Tube 6	Tube 7	Tube 8	Tube 9
Dilution	0.19 μl/500 ml	0.39 μl/500 ml	0.78 μl/500 ml	1.56 μl/500 ml	3.123 μl/500 ml	6.25 μl/500 ml	12.5 μl/500 ml	25 μl/500 ml	50 μl/500 ml
Add *Candida *emulsion	500 μl of emulsion	500 μl of emulsion	500 μl of emulsion	500 μl of emulsion	500 μl of emulsion	500 μl of emulsion	500 μl of emulsion	500 μl of emulsion	500 μl of emulsion
Final dilution	0.09 μl/ml	0.19 μl/ml	0.39 μl/ml	0.78 μl/ml	1.56 μl/ml	3.123 μl/ml	6.25 μl/ml	12.5 μl/ml	25 μl/ml

The MIC and MFC of both essential oils were determined individually and in combination to assess the antifungal activity against *C. albicans*.

Determination of synergistic effects of essential oil using the fractional inhibitory concentration index (FICI)

In order to determine the synergistic activity of essential oils, FICI was used [18].

$FICI = MICA in combination/MICA alone + MICB in combination/MICB alone$

where MICA is the MIC of *O. vulgare* oil and MICB is the MIC of *S. aromaticum* oil (Table 2).

**Table 2:** FICI FICI: fractional inhibitory concentration index

FICI	Index
< 0.5	Synergism
> 0.5 - 1	Additive effects
> 1 - < 4	Indifference
> 4	Antagonism

Preparation of antifungal herbal gel containing *O. vulgare* and *S. aromaticum* essential oils

The antifungal herbal gel was performed by dispensing Carbopol 940 in 5 ml of distilled water with constant stirring and was left overnight to enhance the process of swelling. Further, ethanol and polyethylene glycol 400 were mixed with the above mixture, followed by the addition of *O. vulgare* and *S. aromaticum* essential oils based on the MIC and MFC obtained, and the total volume was made up to 10 ml by adding the remaining part of distilled water. All the components were mixed uniformly with Carbopol 940 to obtain the smooth consistency of the antifungal herbal gel. Finally, different formulations of the gel were prepared (Table 3).

**Table 3:** Composition of herbal antifungal gel formulations *O. vulgare*: *Origanum vulgare*,* S. aromaticum*: *Syzygium aromaticum*

S. No	Ingredients	Formulation 1	Formulation 2	Formulation 3
1	*O. vulgare* oil	2 μl	4 μl	6 μl
2	*S. aromaticum* oil	2 μl	4 μl	6 μl
3	Carbopol 940	0.15 gm	0.15 gm	0.15 gm
4	Ethanol	0.4 ml	0.4 ml	0.4 ml
5	Polyethylene glycol 400	0.5 ml	0.5 ml	0.5 ml
6	Distilled water	10 ml	10 ml	10 ml

Evaluation of physical properties of the formulated herbal gel

The formulated gel was assessed for visual appearance (color, homogeneity, consistency), smell, and tactile sensation. The viscosity of the antimicrobial gel was assessed using a Brookfield viscometer. The pH was determined by a pH meter. The spreadability of the gel was calculated by using the formula:

$S = M x L / T$

where M is the weight tied to the upper slide, L is the length of the glass slides, and T is the time taken to separate the slide.

Determination of antifungal activity of the formulated herbal gel

The antifungal activity test was performed using standard *C. albicans* strains (ATCC 90028). Sterile SDA culture plates were prepared and kept aside for drying and cooling. Inoculation of *C. albicans* was done by streaking using a micron wire loop. A sterile cork borer 6 mm in diameter was used to punch holes about 4 mm deep, and 0.5 ml of gel from each formulation was inoculated. The culture plates were then incubated at 37°C for 48 hours. Then, the inhibition zone (diameter in mm) was measured using a thin, transparent millimeter scale in comparison with a standard antifungal disc.

Cytotoxicity assay

Cell Proliferation/Cytotoxicity Assay

The 3-(4,5-dimethylthiazol-2-yl)-2,5-diphenyltetrazolium bromide (MTT) assay was performed on L929 mouse embryonic fibroblasts. Cell lines of L929 were procured from the National Center for Cell Sciences, Pune, with a detailed datasheet including Short Tandem Repeat (STR) profiling with 100% matching with ATCC cell lines. After procuring the cell lines, maintenance and subculturing of the cells were done by preparing 100 ml of complete media comprising Dulbecco's Modified Eagle's Medium 89 ml (Himedia, Ref: AL250A), fetal bovine serum 10 ml (Himedia RM 10432, LOT 573421), and antibiotics 1 ml (Himedia A002, LOT 5392281).

The cells were maintained in a 5% carbon dioxide incubator and observed under an inverted light microscope. All the procedures for cell culture were performed in the Class II cabinet by considering all the aseptic conditions. On cells reaching 85% confluency, trypsinization was performed using trypsin (TCL007, LOT 536691), and subculturing was done as per the standard protocol. A trypan blue dye exclusion assay was done to determine and calculate the number of viable cells.

Day 1 Cell Seeding

During the log phase of growth, the MTT assay was performed on the L929 cells. The assay was designed by considering negative and positive controls. The MTT assay was performed using 96 treated flat-bottom well plates. The markings were done on 96-well plates by considering negative control (without adding gel), positive control (standard antifungal gel), and herbal antifungal gel of 100%, 50%, 25%, 12.5%, 6.25%, and 3.12%. The assay was set with compounds in triplicate wells to avoid any bias. A trypan blue assay was performed, and the number of L929 viable cells was counted in the Neubauer counting chamber and calculated. Then 10000 cells were seeded per well in a 96-well plate. Later, complete media was added to make a volume of 150 μl and incubated for 24 hours in a 5% carbon dioxide incubator.

Day 2 Compound Treatment

After 24 hours of incubation, once the cells were attached to the plate, the cells were treated with all the concentrations of herbal antifungal gel: 100%, 50%, 25%, 12.5%, 6.25%, and 3.12%, and further incubated for 24 hours in a 5% carbon dioxide incubator.

Day 3 MTT Dye Treatment

The superficial media of cells treated with the compound was removed and thoroughly washed twice with phosphate-buffered saline. MTT dye of 20 μl was added to each well, and the plate was wrapped with silver foil as MTT dye is photosensitive and incubated for four hours. The supernatant from all the wells was slowly removed and discarded without disturbing the formed formazan crystals. DMSO of 100 μl was added to all the wells to dissolve the formazan crystals, and a spectrophotometer reading of around 570 nm was obtained. The proliferative index was calculated by dividing the optical density (OD) of the test with the OD of the control multiplied by 100.

## Results

**Figure 2:**
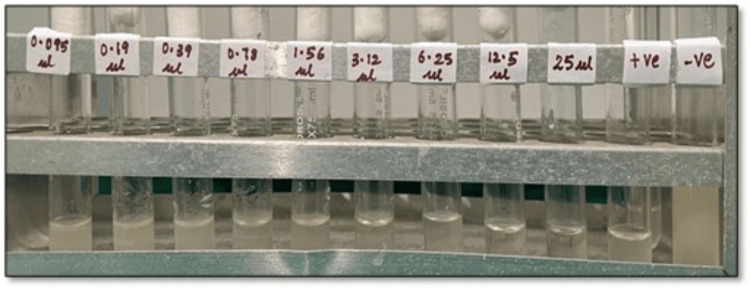
Photomicrograph showing the MIC of O. vulgare essential oil serially diluted with 1% DMSO, ranging from 0.095 μl/ml to 25 μl/ml, along with a positive and negative control. The first four tubes show turbidity, which denotes the presence of C. albicans, and clearing was observed in the fifth tube with a concentration of 1.56 μl/ml, which denotes the fungistatic action of O. vulgare oil on C. albicans inoculum MIC: minimum inhibitory concentration, DMSO: dimethyl sulfoxide, *C. albicans: Candida albicans*, *O. vulgare*: *Origanum vulgare*

The MIC and MFC of *O. vulgare *and *S. aromaticum* essential oils individually and in combination were done to assess the synergistic activity against *C. albicans*. Based on our study, we found that the MIC was 1.56 μl/ml and the MFC was 3.12 μl/ml for *O. vulgare* essential oil, respectively (Figure 2).

**Figure 3:**
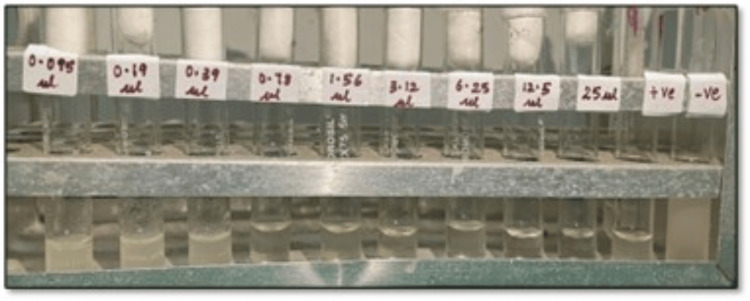
Photomicrograph showing the MIC of S. aromaticum essential oil serially diluted with 1% DMSO, ranging from 0.095 μl/ml to 25 μl/ml, along with a positive control and a negative control. The first three tubes show turbidity, which denotes the presence of C. albicans, and clearing was observed in the fourth tube with a concentration of 0.78 μl/ml, which denotes the fungistatic action of S. aromaticum oil on C. albicans inoculum MIC: minimum inhibitory concentration, DMSO: dimethyl sulfoxide, *C. albicans: Candida albicans, S. aromaticum: Syzygium aromaticum*

The MIC for *S. aromaticum* essential oil was found to be 0.78 μl/ml, and the MFC was 1.56 μl/ml (Figure 3).

**Figure 4:**
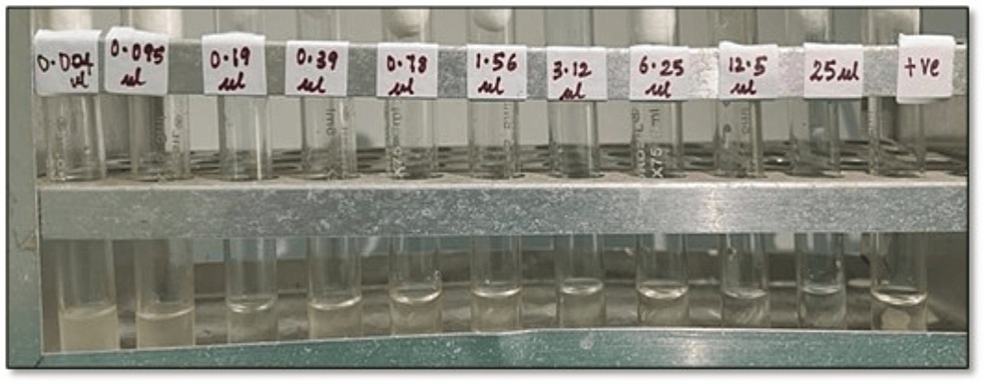
Photomicrograph showing the MIC of S. aromaticum and O. vulgare essential oils in combination with an equal quantity that is serially diluted with 1% DMSO, ranging from 0.004 μl/ml to 25 μl/ml, along with a positive control. The first two tubes show turbidity, which denotes the presence/growth of C. albicans, and clearing was observed in the third tube with a concentration of 0.19 μl/ml, which denotes the fungistatic action of a combination of S. aromaticum and O. vulgare essential oils on C. albicans inoculum MIC: minimum inhibitory concentration, DMSO: dimethyl sulfoxide, *C. albicans: Candida albicans*,* O. vulgare*:* Origanum vulgare*, *S. aromaticum: Syzygium aromaticum*

We also found that a combination of *O. vulgare* and *S. aromaticum* essential oils showed antifungal activity at a lesser concentration, with a MIC of 0.19 μl/ml and MFC of 0.39 μl/ml when compared to their individual concentrations (Figure 4, Table 4).

**Table 4:** MFC of essential oil against C. albicans MFC: minimum fungicidal concentration, *C. albicans: Candida albicans, *ATCC: American Type Culture Collection, *O. vulgare*: *Origanum vulgare,* *S. aromaticum*: *Syzygium aromaticum*

MFC of essential oil against *C. albicans*									
Organism	Essential oil	0.09 µl	0.19 µl	0.39 µl	0.78 µl	1.56 µl	3.12 µl	6.25 µl	12.5 µl
*Candida albicans* ATCC (90028)	*O. vulgare* oil	Growth seen	Growth seen	Growth seen	Growth seen	Growth seen	No growth	No growth	No growth
	*S. aromaticum* oil	Growth seen	Growth seen	Growth seen	Growth seen	No growth	No growth	No growth	No growth
	Combination of *O. vulgare* and *S. aromaticum* oils	Growth seen	Growth seen	No growth	No growth	No growth	No growth	No growth	No growth

Furthermore, we determined the synergistic effect using the FICI index value, which was around 0.3. On physical evaluation, the prepared gels in different concentrations were glassy white in color, smooth in consistency, and had an aromatic odor. The pH of the formulated gel was adjusted to 7.3-7.4, compatible with the oral mucosal surfaces. The viscosity of all the formulations was around 796.6 + 1.76 poise. The prepared gels in different concentrations were spreadable, and formulation three showed a maximum spreadability of around 6.4 cm, followed by formulation two and formulation one, which were around 5.8 cm and 5.6 cm, respectively.

**Figure 5:**
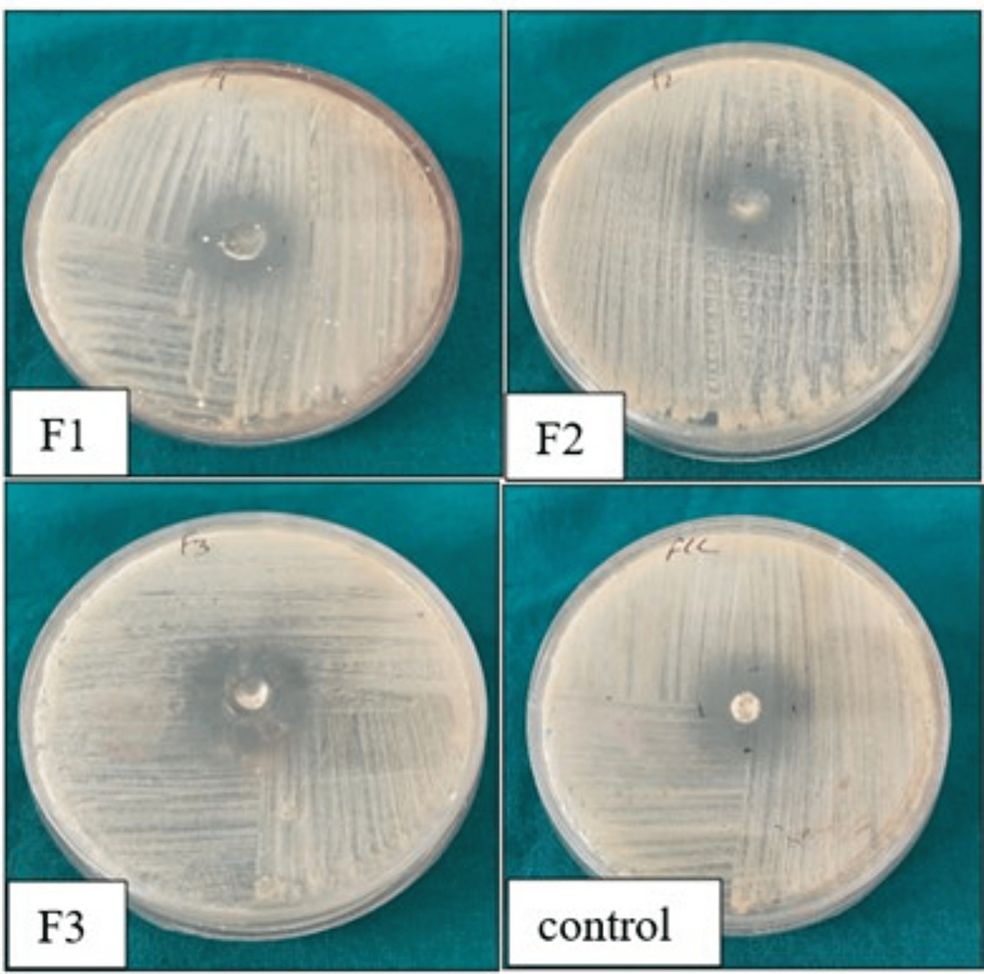
Photomicrograph showing zone of inhibition of the formulated gel (F1, F2, and F3) with a standard antifungal disc (fluconazole)

The antifungal activity of the prepared herbal gel was determined by the zone of inhibition in comparison with a standard antifungal disc (fluconazole). We found that formulation three (F3) with a concentration of 0.6 μl/ml of *O. vulgare *and *S. aromaticum* essential oils has shown antifungal activity equal to that of standard antifungal discs at a very low concentration, with a zone of inhibition of around 19 mm (Figure 5).

**Figure 6:**
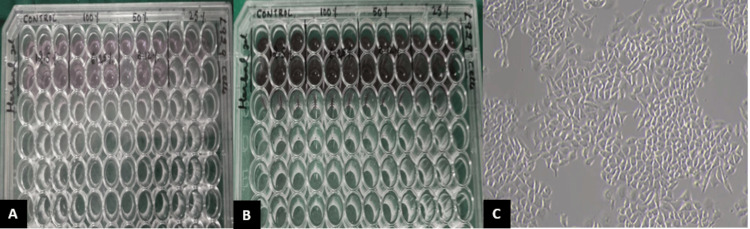
Photomicrograph of the cytotoxicity test - MTT assay (A) MTT assay of L929 cells from 10000 cells was seeded in each 96-well plate. (B) MTT assay of L929 cells showing no toxicity at all concentrations with dissolved purple formazan in herbal gel when compared to negative and positive controls. (C) MTT assay of L929, mouse embryonic fibroblasts MTT: 3-(4,5-dimethylthiazol-2-yl)-2,5-diphenyltetrazolium bromide

Further, the biocompatible nature of the gel was determined by the MTT assay, and we found that at all concentrations, the herbal antifungal gel did not show any cytotoxicity on L929 mouse fibroblast cells, even compared with the negative control (without adding any gel) and also the positive controls (standard antifungal gel) (Figure 6, Table 5).

**Table 5:** Inference from the MTT assay MTT: 3-(4,5-dimethylthiazol-2-yl)-2,5-diphenyltetrazolium bromide

MTT assay: herbal antifungal gel		
Compound	Concentration of compound (%)	Viability (%)
Herbal gel (0.6 μl/ml)	100	101
Herbal gel (0.6 μl/ml)	50	100
Herbal gel (0.6 μl/ml)	25	91
Herbal gel (0.6 μl/ml)	12.5	90
Herbal gel (0.6 μl/ml)	6.25	85
Herbal gel (0.6 μl/ml)	3.12	80
Positive control (standard antifungal gel)	100	100
Negative control	Without compound	100

Based on our study, we found that an antifungal gel formulation comprising 0.6 μl/ml concentration of *O. vulgare *and *S. aromaticum* essential oils helps to achieve synergistic antifungal activity against oral *C. albicans*.

## Discussion

Despite the development of various standard antifungal agents, the presence of numerous drug-resistant strains and side effects such as drug intolerance and toxicity pose a new challenge; therefore, new therapeutic strategies are necessary to control the fungal infection [19]. Therefore, herbal essential plant oils are emerging as an alternative to antifungal agents as they possess antimicrobial properties due to their accumulation in the cell lipid bilayer, which allows easier transfer and causes cell lysis [20].

Among that, *O. vulgare* is an aromatic herb that has phenolic compounds in its composition, such as carvacrol, thymol and terpin, and α-terpinol and γ-terpene. They directly inhibit germination and hypha formation in *Candida* [21]. There are few studies existing in the literature that have shown the higher antifungal activity of *O. vulgare* oil in comparison to standard antifungal drugs. These studies suggested that *O. vulgare* oil can be replaced with a variety of conventional synthetic antifungal drugs for the treatment of candidiasis [17,22]. However, these studies showed a wide range of *O. vulgare* oil concentrations inhibiting *C. albicans*. In this study, *O. vulgare *and *S. aromaticum *essential oils were evaluated for their antifungal activity against oral *C. albicans*. Various studies have reported that *O. vulgare* and *S. aromaticum* have been extensively used as antimicrobial agents [16,21].

However, on review of existing literature, the synergistic antifungal activity of *O. vulgare* and *S. aromaticum* essential oils against *C. albicans* remains unexplored. In our study, we have shown that a minimal concentration of *O. vulgare* oil with a MIC of 1.56 μl/ml and a MFC of 3.12 μl/ml is required to achieve antifungal activity against *C. albicans*.

Najla et al. showed that the MIC and MFC values of *S. aromaticum* oil were 1.25 μl/ml and 2.5 μl/ml, respectively, against *C. albicans* isolates obtained from systemic candidiasis. These results demonstrated that *S. aromaticum* essential oil was more effective than the anti-mycotic synthetic drug [23]. Pinto et al. conducted a study on oral candidal isolates and have shown that *S. aromaticum* oil exhibited antifungal activity with inhibition of germ tube formation and a MIC range of about 0.64 μl/ml [24]. These results were in accordance with our study, which has shown the antifungal activity of *S. aromaticum* oil at a very low concentration, with the MIC being 0.78 μl/ml and the MFC being 1.56 μl/ml.

Combinations of essential oils produce a synergistic effect that enables potent antifungal action at sufficiently low doses of the active ingredients of the essential oil, which could lead to decreased side effects and also help to overcome the problem of drug resistance [24]. We achieved antifungal activity at a lesser concentration, with a MIC of 0.19 μl/ml and MFC of 0.39 μl/ml when compared to their individual concentrations. Thus, the synergistic effect helps us obtain effective treatment with improved intrinsic activity and relatively less toxicity [25].

There are no studies in the literature regarding the synergistic activity of *O. vulgare* and *S. aromaticum* oil exhibiting antifungal activity with a minimum concentration. This is the first study of its kind that has shown effective antifungal activity against *C. albicans* with a synergistic combination of *O. vulgare *and *S. aromaticum* essential oils.

Only a few studies have shown the antifungal activity of gels containing herbal extracts/essential oils for the treatment of candidiasis [26,27]. To date, there are no commercially available antifungal gels containing *O. vulgare* and *S. aromaticum* essential oils for the management of oral candidiasis. So, we further formulated a gel using a combination of *O. vulgare* and *S. aromaticum* essential oils and showed that with a minimal concentration of 0.6 μl/ml of essential oils, antifungal activity can be achieved compared to the standard antifungal drugs.

The cytotoxic effect of the prepared antifungal gel was evaluated, and the results showed that the gel was biocompatible and did not show any cytotoxic effects, even from the maximum concentration (100%) to the minimum concentration (3.12%).

The limitation of the present study was that the evaluation of the synergistic effect of the two essential oils included in the study was performed in an in vitro setup. The herbal gel was formulated based on its antifungal effect against standard strains of *C. albicans*. However, further studies to determine their MIC and MFC on larger samples of oral isolates will be carried out in the future. Also, the application of the formulated herbal gel in patients for the management of oral candidiasis will be performed through clinical trials, and the antifungal efficacy of the formulated herbal gel will be evaluated and compared with the standard antifungal gel.

## Conclusions

In our present study, we formulated and developed a novel antifungal herbal gel comprising an optimized concentration of 0.6 μl/ml of *O. vulgare *and *S. aromaticum* essential oils to treat oral candidiasis. The formulated gel revealed an antifungal efficacy equal to that of the standard antifungal drug, with a zone of inhibition of around 19 mm. The cytotoxicity/biocompatibility test by MTT assay also revealed that all concentrations of herbal antifungal gel did not show cytotoxicity, thus demonstrating the biocompatible nature of the gel. This gel aids in overcoming the side effects of commercially available standard antifungal drugs, such as drug toxicity and resistance. However, further clinical studies have to be carried out to evaluate the effectiveness of this gel for oral candidiasis management and to assess the patients for improvement in clinical signs and symptoms.
